# The first non Clostridial botulinum-like toxin cleaves VAMP within the juxtamembrane domain

**DOI:** 10.1038/srep30257

**Published:** 2016-07-22

**Authors:** Irene Zornetta, Domenico Azarnia Tehran, Giorgio Arrigoni, Fabrizio Anniballi, Luca Bano, Oneda Leka, Giuseppe Zanotti, Thomas Binz, Cesare Montecucco

**Affiliations:** 1 Department of Biomedical Sciences, University of Padova, Via Ugo Bassi 58/B, Padova, Italy; 2 Proteomics Center, University of Padova and Azienda Ospedaliera di Padova, Padova, Italy; 3 National Reference Center for Botulism, Department of Veterinary Public Health and Food Safety, Istituto Superiore di Sanità (ISS), Roma, Italy; 4 Microbiology and Diagnostic Laboratory, Istituto Zooprofilattico Sperimentale delle Venezie (IZSVe), Vicolo Mazzini 4, Villorba di Treviso, Italy; 5 Medizinische Hochschule Hannover, Institut für Physiologische Chemie OE4310, Hannover, Germany

## Abstract

The genome of *Weissella oryzae* SG25T was recently sequenced and a botulinum neurotoxin (BoNT) like gene was identified by bioinformatics methods. The typical three-domains organization of BoNTs with a N-terminal metalloprotease domain, a translocation and a cell binding domains could be identified. The BoNT family of neurotoxins is rapidly growing, but this was the first indication of the possible expression of a BoNT toxin outside the *Clostridium* genus. We performed molecular modeling and dynamics simulations showing that the 50 kDa N-terminal domain folds very similarly to the metalloprotease domain of BoNT/B, whilst the binding part is different. However, neither the recombinant metalloprotease nor the binding domains showed cross-reactivity with the standard antisera that define the seven serotypes of BoNTs. We found that the purified *Weissella* metalloprotease cleaves VAMP at a single site untouched by the other VAMP-specific BoNTs. This site is a unique Trp-Trp peptide bond located within the juxtamembrane segment of VAMP which is essential for neurotransmitter release. Therefore, the present study identifies the first non-Clostridial BoNT-like metalloprotease that cleaves VAMP at a novel and relevant site and we propose to label it BoNT/Wo.

Botulinum neurotoxins (BoNTs) form a large and growing family of protein neurotoxins that cause the peripheral neuroparalysis of botulism^1^,^2^. These neurotoxins are the most poisonous substances known (50% lethal dose in the range of 0.02 to 1 ng/Kg in laboratory mice) and, accordingly, they are included in the CDC list A as potential bioterrorist agents^3^. This toxicity results from their neurospecific binding and their capacity of entering nerve terminals where they display a metalloprotease activity specific for the three SNARE proteins. Such proteolysis prevents the function of the SNARE nanomachine that mediates the release of neurotransmitters with a consequent prolonged neuroparalysis^1^,^4^,^5^,^6^.

Only bacteria of the genus *Clostridium* have been so far reported to produce neurotoxic BoNTs. The number of different BoNTs is rapidly growing owing to improved DNA sequencing and they are classified in seven distinct serotypes, labeled with letters from A to G, and a progressive number indicating a newly determined amino acid sequence within a serotype^2^,^6^. All BoNTs are capable of performing several biological actions strictly related to the physiology of vertebrate neurons. Indeed, their initial binding to the presynaptic membrane is followed by internalization within acidic organelles wherefrom they translocate their metalloprotease domains into the cytosol; here they cleave specifically the three SNARE proteins which are core components of the nanomachine of neurotransmitter release^6^. This elaborate mechanism of action results from the structural organization of the BoNTs into three domains endowed with specific functions. The N-terminal 50 kDa domain is a metalloprotease that is linked to a central 50 kDa domain (HN) involved in membrane translocation which is followed by the C-terminal domain (HC, 50 kDa) responsible for the binding to nerve terminals^6^,^7^,^8^,^9^,^10^,^11^,^12^. One characteristic feature of the BoNT metalloproteases is their specificity for the three SNARE proteins. In particular, BoNT/B, /D, /F and /G cleave VAMP at different peptide bonds, BoNT/A, /C and /E cleave SNAP-25 and BoNT/C also hydrolyses syntaxin^4^,^10^. In any case, their intracellular activity leads to a long lasting, but reversible, paralysis. These properties are at the basis of the use of BoNT/A1 and BoNT/B1 to treat many human syndromes characterized by hyperfunction of peripheral nerve terminals as the local injection of minute doses of the toxins reverts to a normal function^13^,^14^,^15^,^16^. A further expansion of the therapeutic use of BoNTs is expected from the discovery or design of novel BoNTs endowed with specific useful properties^17^.

Very recently the complete genome of *Weissella oryzae* SG25T, a facultative anaerobe isolated from fermenting rice, an ecological niche that is shared by anaerobic Clostridia, has been determined^18^. Members of the genus *Weissella* are widely distributed in meat, fermented vegetables and soil. Some species have been identified as opportunistic pathogens, some others were proposed as probiotics^19^,^20^,^21^. The bioinformatics analysis of *W. oryzae* SG25T has led to the surprising identification of an open reading frame 1 (*orf1*) that has a strong sequence similarity with *bont* genes, but lacks the additional genes usually associated within the *bont* locus in *Clostridia*^2^,^22^.

## Results

### The BoNT-like protein of *Weissella oryzae* is structurally similar to BoNTs, but does not belong to any known serotype

**Figure 1:**
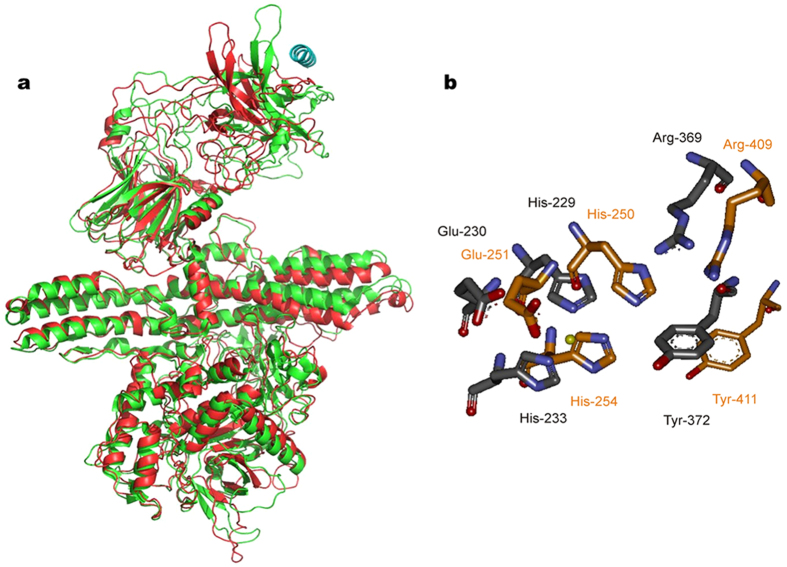
Molecular models of BoNT/B and of the *Weisella oryzae* open reading frame 1 and comparison of their catalytic sites. (**a**) Superposition of the Cα chain trace of BoNT/B (green) to the model of *Weissella oryzae* (red, NCBI gi: 653854119) after molecular dynamics minimization. The structure of the N- and the middle domains, LC and HN respectively, are very similar whilst major differences are observed in the area of the C-terminal domain, HC, where the binding to the polysialoganglioside and synaptotagmin receptor takes place. The α-helix on the top right position (in blue) is the segment 40–60 of synaptotagmin which is involved in BoNT/B binding. (**b**) Arrangement of active site amino acids of BoNT/B (carbon atoms in grey) and comparison with the predicted configuration of corresponding residues of Wo-ORF1 (carbon atoms in orange). In addition to the presence of the zinc-binding motif residues of metalloproteases, His-Glu-X-X-His, also the Arg and Tyr residues (residues 369 and 372 of BoNT/B) of the second shell of zinc coordination are present and show the same configuration. The yellow sphere represents the active site zinc atom.

Given the paramount and multifaceted importance of BoNTs, we decided to test whether the BoNT-like *orf1* gene of *Weissella oryzae* indeed codes for a metalloprotease similar to the LC of BoNTs. We chose BoNT/B for a structural comparison, given the higher resolution of its crystallographic structure. A molecular model was built using the crystal structure of BoNT/B complexed with the segment 40–60 of its cellular protein receptor synaptotagmin (PDB ID Code 2NP0)^23^. After minimization, a molecular dynamics simulation (using software package GROMACS ver. 4.6: http://www.gromacs.org.)^24^ was run for 20 ns and the final result is shown in Fig. 1a. The LC and the central HN domains of the Wo-ORF1 protein fold very similarly to the corresponding ones of BoNT/B. Figure 1b shows that, in addition to the presence of the zinc-binding motif of metalloproteases, the LC of Wo-ORF1 protein also contains the Arg and Tyr residues (residues 369 and 372 of BoNT/B) of the second shell of zinc coordination that are unique of the clostridial metalloproteases family and of the anthrax lethal factor^10^,^25^. These residues are essential for the metalloprotease activity of the BoNT and their possible role in catalysis has been proposed^10^,^26^,^27^. Also the C-terminal domains present an overall similarity in their first half. Significantly, the second half, which is the part of BoNT/B responsible for neurospecific binding to the oligosaccharide portion of polysialogangliosides and to the α-helical binding segment 47–58 of synaptotagmin, is different suggesting a peculiar binding specificity of the Wo-ORF1 protein. None of the BoNT/B residues forming the hydrophobic saddle-like binding area for synaptotagmin, like Trp-1178, Tyr-1181, Tyr-1183, Phe-1194, Phe-1204, are conserved, nor the Wo-ORF1 protein presents the BoNT binding pocket for polysialogangliosides which is characterized by the presence of the Ser-X-Trp-Tyr…Gly motif^12^.

**Figure 2:**
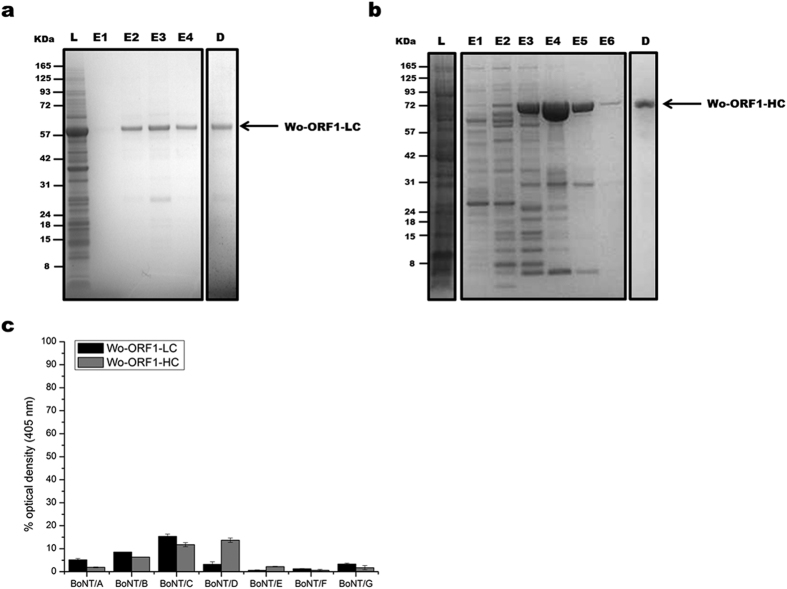
Purification of recombinant Wo-ORF1 proteins and serotyping by indirect ELISA. Panels (**a**,**b**) show, respectively, the purification and characterization of Wo-ORF1-LC and of Wo-ORF1-HC, as recombinant proteins. Full-length Wo-ORF-LC or Wo-ORF1-HC (arrows) were expressed in competent *E. coli* cells. Total lysates “L” were purified with an affinity HisTrap HP column. The eluted fractions “E”, followed by progressive numbers, were pooled and dialysed overnight “D”. SDS-PAGE gel was stained with SimplyBlue™ SafeStain. These images are representative of three independent expression and purifications experiments. (**c**) The seven BoNT serotypes or Wo-ORF1-LC or Wo-ORF1-HC were incubated with BoNT serotype specific polyclonal antisera provided by the CDC, Atlanta. The ELISA test was developed using ABTS. The mean optical density at 405 nm of the average of three independent sets of experiments each one consisting of triplicates. The values obtained with the standard sera (anti serotype A, BoNT/A; anti serotype B, BoNT/B; etc.) were taken as 100% and the values obtained with the *Weissella* proteins expressed as percentages. Bars represent S.D. values.

The light chain (Wo-ORF1-LC) and the C-terminal domain (Wo-ORF1-HC) of the Wo-ORF1 protein were produced as recombinant proteins in *E. coli* (Fig. 2, panels a and b, respectively). This approach is based on the large body of literature proving that the recombinant domains of BoNTs preserve the biological properties they have in the holotoxin. Their immunoreactivity was tested by indirect ELISA using the seven CDC standard BoNT antisera that are employed to define the seven BoNT serotypes^28^. None of them recognized the recombinant Wo-ORF1-LC and the -HC domains, except for a weak cross-reaction with the anti-BoNT/C and the anti BoNT/D antisera (Fig. 2c).

### The BoNT-like protein of *Weissella oryzae* is a novel metalloprotease that cleaves VAMP

**Figure 3:**
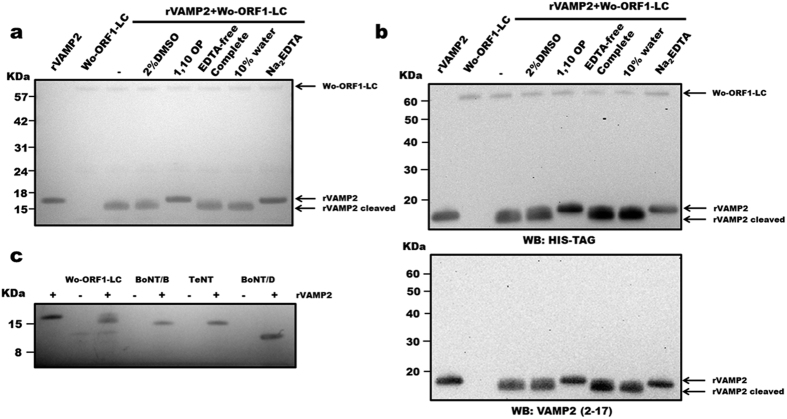
Metalloproteolytic activity of Wo-ORF1-LC and comparison with those of Clostridial neurotoxins. (**a**) 1 μg of rVAMP2 was incubated with 1 μg of recombinant Wo-ORF1-LC in 50 mM Na_2_HPO_4_, pH 7.4 in the presence or absence of different inhibitors or their vehicles, as indicated. SDS-PAGE gel was stained with SimplyBlue™ SafeStain. (**b**) The same samples described in panel (**a**), were immunoblotted using an anti-His tag antibody (top panel) or an antibody recognizing the N-terminal segment of VAMP2 (bottom panel). (**c**) 1 μg of rVAMP2 was incubated with 1 μg of recombinant Wo-ORF1-LC or BoNT/B, /D and TeNT in 50 mM Na_2_HPO_4_ buffer, pH 7.4, as indicated. SDS-PAGE gel was stained with SimplyBlue™ SafeStain. All figures are representative at least of three independent experiments.

The possibility that Wo-ORF1-LC acts like the LC of BoNTs was tested using an enzymatic assay established for these neurotoxins^29^. Figure 3a shows that the cytosolic domain of rat VAMP2 (segment 1–97), which is commonly used for this assay, is cleaved by Wo-ORF1-LC with the formation of a large fragment, approximately one thousand daltons (Da) smaller than the intact substrate. No cleavage was detected in the presence of EDTA or 1,10-phenanthroline, which specifically inhibit metalloproteases by chelating the active site zinc ion (Fig. 3a). On the contrary, the same cleavage was observed in the presence of a complete protease inhibitor EDTA-free cocktail, capable of blocking the activity of a large spectrum of serine and cysteine proteases, leaving the function of metal-dependent proteases unaffected (Fig. 3a). Furthermore, the proteolysis of rat VAMP2 likely occurred within the C-terminus retaining both the N-terminal histidine tag used for purification and the segment 2–17 of rat VAMP2 (Fig. 3b top and bottom panels, respectively). This finding and a comparison with the cleavages by the TeNT, BoNT/B and /D (Fig. 3c) indicates that Wo-ORF1-LC cleaves VAMP2 close to its C-terminus.

### VAMP2 is cleaved within its juxtamembrane segment at a unique and essential W-W peptide bond

**Figure 4:**
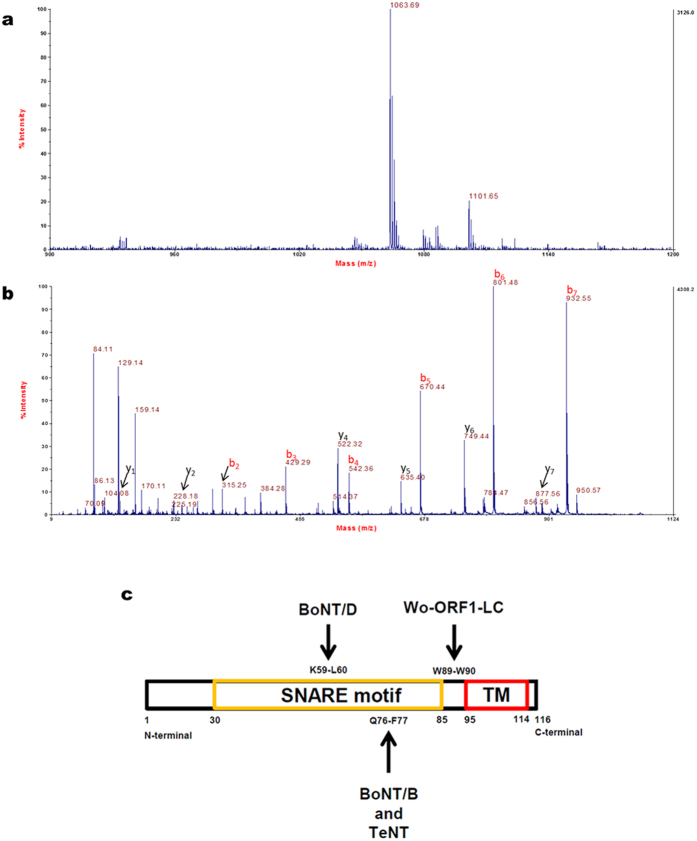
Identification of the cleavage site using mass spectrometry analysis. (**a**) MS spectrum acquired in reflector mode of the peptide at m/z = 1063.69 Da, which is generated upon incubation of rVAMP2 with Wo-ORF1-LC in the absence of EDTA. (**b**) Annotated MS/MS spectrum of the peptide at m/z = 1063.69 Da. b and y ion series are almost complete and indicate that the peptide sequence is WKNLKMMI (theoretical monoisotopic mass 1063.56 Da; 122 ppm mass difference). The mass spectrometry analysis was performed twice and representative spectra are shown. (**c**) Localization and relative length of the SNARE domain, the juxtamembrane region and the transmembrane (TM) domain of VAMP with the cleavage sites of the proteases used in Fig. 3c.

In order to determine the exact site(s) of VAMP2 proteolysis, the recombinant rat VAMP2 1–97 (molecular mass 12668.7 Da) was incubated with Wo-ORF1-LC in the presence (Fig. S1a) or absence of EDTA (Fig. S1b) and subjected to mass spectrometry analysis. The Wo-ORF1-LC metalloprotease generated a fragment of 11623.4 Da (Fig. S1b) and a smaller one of 1063.69 Da (Fig. 4a). The sequence of the latter one was determined by MS/MS analysis to be WKNLKMMI (Fig. 4b). Therefore, Wo-ORF1-LC cleaves VAMP2 at a single peptide bond which is Trp89-Trp90. This is a novel cleavage site compared to BoNTs and it is located within the juxtamembrane segment of VAMP (Fig. 4c). The proteolysis of VAMP by Wo-ORF1-LC releases the entire cytosolic domain of VAMP from the synaptic vesicle membrane and one can predict that this will prevent the assembly of the trans-SNARE complex on the membrane and the ensuing vesicle-target membrane fusion^4^,^5^,^6^.

### The VAMP W-W peptide bond is highly, but not completely, conserved

The W-W motif has been shown to be essential for the process of neurotransmitter release^30^,^31^,^32^,^33^. The W-W peptide bond is present in several VAMPs (Fig. S2a) whilst in other VAMPs the P1′ site is occupied by a range of different residues and rat VAMP5 contains an Arg residue in the P1 position. We cannot predict whether these different VAMPs will be cleaved by Wo-ORF1-LC. The neuronal VAMP of *Drosophila simulans* contains the W-L peptide bond and it is not cleaved by Wo-ORF1-LC, whilst it is cleaved by the LC of tetanus neurotoxin^34^ (Fig. S2b).

## Discussion

The general and relevant conclusion of the present work is that the putative BoNT-like LC encoded by *Weissella oryzae* SG25T is indeed a metalloprotease similar to the *Clostridial* botulinum neurotoxins. This conclusion is based on multiple evidence. First, a high structural similarity of the *W. oryzae* BoNT-like LC sequence which includes the unique active site of the BoNT family of metalloproteases. Second, we determined that the *W. oryzae* BoNT-like LC protein cleaves VAMP2 like tetanus toxin and four BoNTs do, but at a single and unique peptide bond: Trp89-Trp90. Thus this protease removes the entire cytosolic domain of VAMP which includes the SNARE domain involved in the formation of the SNARE complex necessary for vesicle-target membrane fusion^4^,^5^,^6^. Previous mutagenesis experiments have clearly shown that this WW-motif is essential for the function of VAMP in the process of neurotransmitter release^30^,^33^. Based on these results and on the finding that there is no reactivity among the N- and C-terminal domains with the standard BoNT serotype specific antisera, we would like to propose that the *orf1* gene of *W. oryzae* codes for a novel toxin that could be termed BoNT/Wo.

Mansfield *et al.*^22^ have suggested that this toxin may interfere with the SNARE-mediated plant defense system. Indeed, plant cells produce defensins containing vesicles whose membrane contains several copies of VAMP protein isoforms termed longins which contain the juxtamembrane W-W peptide bond^35^ (Fig. S2a). However, considering the ecological niche where *W. oryzae* proliferates, we would like to propose that it is also possible that BoNT/Wo acts on worms or amoebe that feed on bacteria to prevent their ingestion and phagocytosis. Indeed, worms and amoebae express VAMP proteins possessing the juxtamembrane W-W peptide bond (Fig. S2a).

The Wo-ORF1 gene is not flanked by any of the accessory proteins that characterize *bont* genes within Clostridia and that encode for proteins that form large progenitor complexes with BoNT^2^,^6^,^22^. Such complexes are involved in the process of uptake of BoNT from the intestinal lumen into the general circulation^6^,^36^. A likely possibility is that *W. oryzae* acquired a portion of the *bont* locus from a neurotoxigenic *Clostridium* bacterium present within the same ecological niche inhabited by *W. oryzae*. More investigations are required to define the biological role of this novel bacterial toxin as well as its evolutionary origin. Finally, further studies are needed to assess *W. oryzae* as a possible emerging agent of foodborne diseases.

## Methods

### Recombinant *Weissella oryzae* Open Reading Frame 1 LC and HC domains

According to NCBI gi: 653854119, the cDNA sequences of Wo-ORF1-LC (residues 1–476) and Wo-ORF1-HC (residues 910–1296) were codon optimized for *E. coli* and synthetized by GeneArt (ThermoFisher Scientific). They were cloned into pRSET A vector (ThermoFisher Scientific), containing polyhistidine sequence, as an BamHI/KpnI insert with a N-terminal c-Myc (EQKLISEEDL) or an BamHI/HindIII insert with a EGFP N-terminal tag, respectively. They were transformed in competent *E. coli* cells (EMD Millipore), and bacteria were grown for 4 hours in the presence of 1 mM IPTG. After centrifugation, cells were lysed and the resulting suspension was sonicated and centrifuged. The soluble portion of Wo-ORF1-HC was subjected to Ni-charged column for affinity chromatography (GE Healthcare) according to manufacturer’s instructions. The insoluble portion of Wo-ORF1-LC was suspended by overnight stirring in the presence of 0.5% n-lauroyl sarcosine. Following centrifugation, the supernatant was purified using the same affinity chromatography. The pooled fractions of Wo-ORF1-LC and -HC were dialysed in 150 mM NaCl, 20 mM Tris-HCl, pH 7.4. All reagents were purchased from Sigma-Aldrich if not specified.

### Indirect ELISA

400 ng/mL of Wo-ORF1-LC or Wo-ORF1-HC or the seven BoNT serotypes were coated overnight onto 96-well-plates in PBS at 4 °C. After standard procedure of blocking and washing, proteins were incubated for 2 h at room temperature with the standard set of CDC polyclonal anti BoNTs, in 0.05% Tween-20, 0.1% BSA in PBS. The ELISA was developed, after 1 hour incubation with HRP-conjugated secondary antibody using 2,2′-Azino-bis(3-ethylbenzothiazoline-6-sulfonic acid). The absorbance value given by the seven BoNT serotypes and their specific CDC reference antiserum were taken as 100% and the values obtained with the *W. oryzae* protein domains were expressed as%values after correction for the different MW of the proteins tested.

### VAMP2 cleavage assay

1 μg of recombinant rat VAMP/synaptobrevin 2 (rVAMP2) 1–97 was incubated with 1 μg of Wo-ORF1-LC or with previously reduced BoNT/B, /D or TeNT (in 50 mM NaHPO_4_, pH 7.4), for 4 hours at 37 °C under stirring. In some samples, the mixtures were incubated in the presence of 10 mM 1,10 ortho-phenantroline monohydrate or 10 mM Na_2_EDTA or Complete^TM^Protease Inhibitor-EDTA free (Roche). The samples were subjected to immunoblotting using specific antibodies for anti-Histidine-Tagged-Antibody (EMD Millipore) or monoclonal mouse antibody against the N-terminus of VAMP2 (Synaptic System). Both the soluble and precipitate fractions of recombinant Wo-ORF1-LC displayed the same metalloprotease activity.

The plasmid encoding for the full length *Drosophila simulans* VAMP (termed nSyb) was provided by Prof. Hiesinger. It was subcloned into pET-28a (Novagen) expression vector between EcoRI/XhoI restriction sites. The construct was then checked by DNA sequencing. *E. coli* BL21 (DE3) were induced with 1 mM IPTG, followed by overnight culture at 16 °C. Purification of nSyb was carried out as described above. The proteolysis tests were carried out as described above in the presence of 0.1% octyl-glucoside to disperse nSyb, as other detergents were found to inactivate Wo-ORF1-LC.

### Mass Spectrometry

RVAMP2 was analyzed by mass spectrometry using a 4800 MALDI-TOF/TOF (AB Sciex). A solution 0.2 μg/μL of rVAMP2 in 5 mM NaHPO_4_, pH 7.4 was used for the analysis of the high molecular weight region (m/z 8000–18000 Da): 1 μL of rVAMP2 (incubated with Wo-ORF1 LC, in presence or absence of 10 mM Na_2_EDTA) was mixed with an equal amount of sinapinic acid (10 mg/mL in 70% acetonitrile/0.1% formic acid; Sigma Aldrich). 0.8 μL of the resulting mix was spotted onto a stainless steel MALDI plate and subjected to MS analysis in linear mode. Acceleration voltage was set to 20 kV and delay extraction to 2200 ns. In total 1500 laser shots were accumulated to obtain the final spectra, with a laser energy of 6000 (arbitrary units). The same solutions of VAMP2 were also used for the analysis of the low molecular weight region (m/z 900–3500 Da): 1 μL of each solution was mixed with 1 μL of α-cyano-4-hydroxycinnamic acid (5 mg/mL in 70% acetonitrile/0.1% formic acid, Fluka). The resulting mix was spotted onto the MALDI plate and analyzed in reflector mode using an acceleration voltage of 20 kV, a grid voltage of 16 kV and a delay extraction of 450 ns, with a laser energy of 3500 (arbitrary units). MS/MS spectra were acquired with a laser energy of 4500 (arbitrary units), by setting 8 kV for source 1 and 15 kV for source 2. Air was used in the collision cell and 3500 laser shots were accumulated to obtain the final spectra. MS/MS spectra were manually interpreted and annotated.

## Additional Information

**How to cite this article**: Zornetta, I. *et al.* The first non Clostridial botulinum-like toxin cleaves VAMP within the juxtamembrane domain. *Sci. Rep.* **6**, 30257; doi: 10.1038/srep30257 (2016).

## Supplementary Material

Supplementary Information
