# Supplementary material for: The first non Clostridial botulinum-like toxin cleaves VAMP within the juxtamembrane domain
Source: Sci Rep. 2016 Jul 22;6:30257. doi: 10.1038/srep30257 (PMC4957215; doi:10.1038/srep30257)
Supplement: Supplementary Information [file srep30257-s1.pdf]

The first non Clostridial botulinum-like toxin cleaves VAMP within the juxtamembrane domain

Irene Zornetta[1,*], Domenico Azarnia Tehran[1,*], Giorgio Arrigoni[1,2,*], Fabrizio Anniballi[3], Luca Bano[4], Oneda Leka[1], Giuseppe Zanotti[1], Thomas Binz[5] and Cesare Montecucco[1]

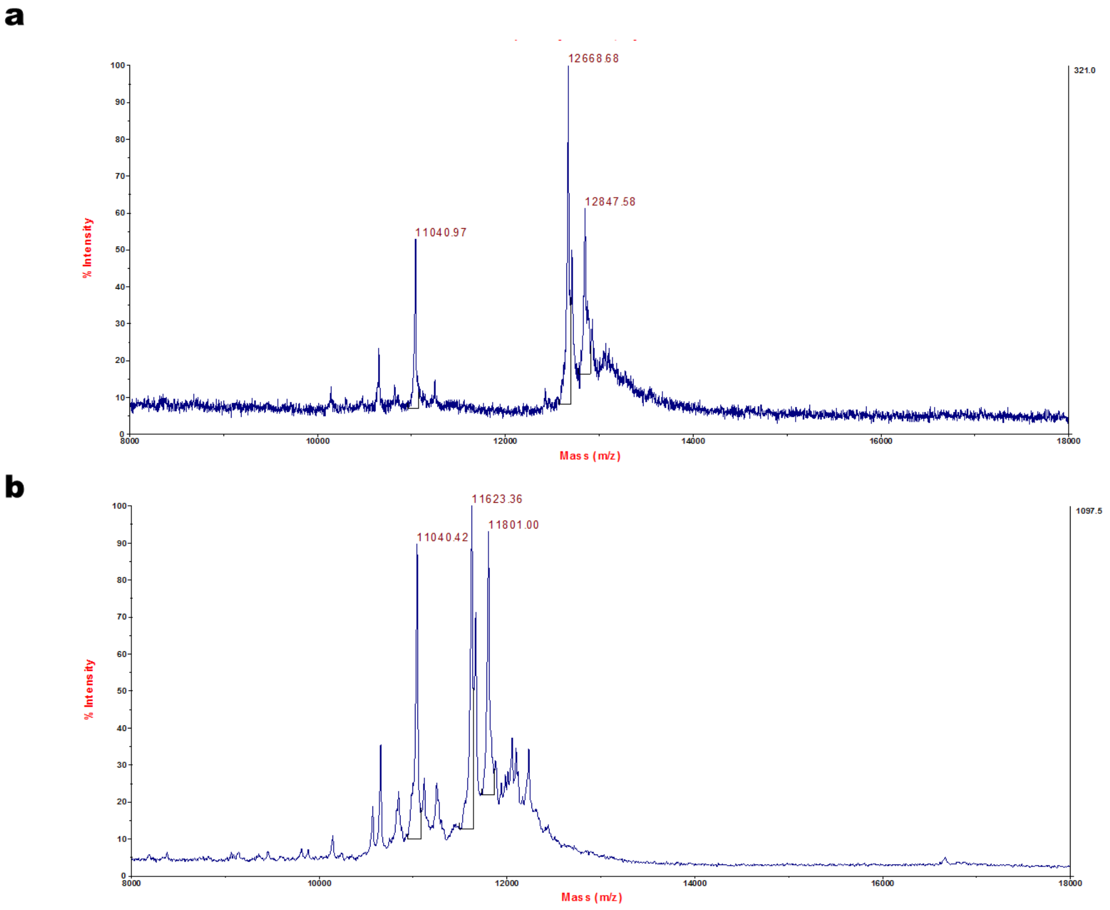

Figure S1. MS spectra of rVAMP2 incubated with Wo-ORF1-LC. MS spectra of rVAMP2 (1-97) acquired in linear mode in the presence (panel a) or absence (panel b) of EDTA. The intact segment 1-97 (m/z 12668.7 Da, panel a) is cleaved by Wo-ORF1-LC with a consequent mass shift at 11623.4 Da (panel b; mass difference of 1045.4 Da). The newly generated peptide (Fig. 4a) was identified with m/z= 1063.69 Da, equivalent to the enzymatic addition of a water molecule to the 1045.4 Da fragment. The peptide sequence was identified by MS/MS as shown in Fig. 4b. The MS spectra analysis was performed twice and representative spectra are shown.

a

```
SP|O02495|SYB1_CAEEL  65  GASQFEKSAA TLKRKYWWKN IKMMIIMCAI VVILIIIIVL WAG  107
SP|Q54NW7|VAM7A_DICDI 168 ESFVFKSNSV ALKRKLWWQN KKLAIAIGLV VCILIAVITL ALLK 210
SP|Q86AQ7|VAM7B_DICDI 168 QSFKFKKQSK QLKCAMWWKN VKLMLVLGAI VLIIIFIIVM SYC  210
SP|O49377|VA711_ARATH 168 NTFRFRKQAR RFRSNVWWRN CKLTVLLILL LLVIIYIAVA FLC  210
SP|Q9SIQ9|VA712_ARATH 168 NTFRFRKQTR RFNNTVWWRN CKLTLLLILV LLVIIYIGVA FAC  210
SP|Q9LFP1|VA713_ARATH 168 NTFRFRKQAR RYRTIMWWRN VKLTIALILV LALVVYIAMA FVC  211
SP|Q63666|VAMP1_RAT   75  GASVFESSAA KLKRKYWWKN CKMMIMLGAI CAIIVVVIVI YI   116
SP|P63045|VAMP2_RAT   73  GASQFETSAA KLKRKYWWKN LKMMIILGVI CAIILIIIIV YF   114
SP|P63025|VAMP3_RAT   60  GASQFETSAA KLKRKYWWKN CKMWAIGISV LVIIVIIIIV WCV  102
SP|Q9WUF4|VAMP8_RAT   53  TSEHFKTTSQ KVARKFWWKN VKMIVIICVI VLIILILIIL FAT  95
TR|A0A0J9RNB2_DROSI   83  GASQFEQQAG KLKRKFWLQN LKMMIIMGVI GLVVVGIIA       121
SP|P18489|SYB_DROME   89  GASQFEQQAG KLKRKQWWAN MKMMIILGVI AVVLLIIVLVSV    130
```

b

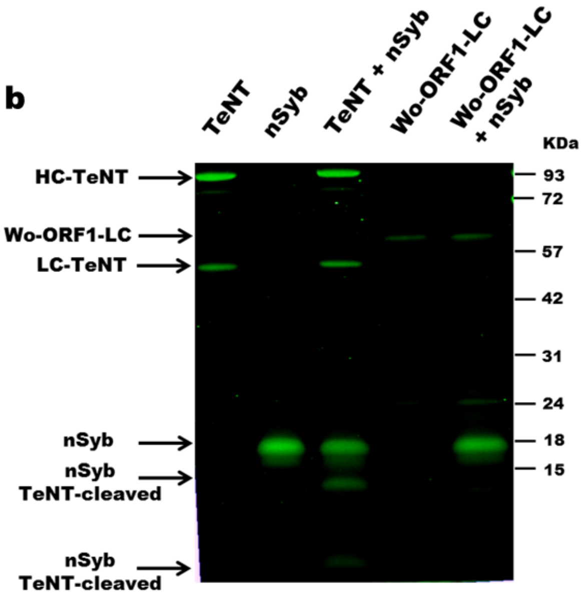

Figure S2. Alignment of the C-terminal part of VAMPs of different organisms and *in vitro* nSyb proteolytic cleavage. For the alignment in (a), we selected the region proximal to the transmembrane, indicated in grey. CAEEL indicates *C. elegans,* DICDI *D. discoideum*, ARATH *A. thaliana*, RAT *R. norvegicus,* DROSI*, D. simulans and* DROME, *D. melanogaster*. (b) 1 µg of nSyb was incubated with 1 µg of Wo-ORF1-LC in 50 mM $NaHPO_4$ buffer supplemented with 0.1% octyl-glucoside, pH 7.4 for 4 hours at 37°C under stirring. SDS-PAGE gel was stained with SimplyBlue™ SafeStain and it was imaged on Odyssey® LI-COR, more sensitive than white-light imaging of Coomassie staining[1]. This image is representative of two independent experiments.

References

1 Luo, S., Wehr, N. B. & Levine, R. L. Quantitation of protein on gels and blots by infrared fluorescence of Coomassie blue and Fast Green. *Anal Biochem* **350**, 233-238 (2006).
